# Structural Damage Identification Using the Optimal Achievable Displacement Variation

**DOI:** 10.3390/ma15238440

**Published:** 2022-11-26

**Authors:** Xi Peng, Cunkang Tian, Qiuwei Yang

**Affiliations:** 1 School of Civil and Transportation Engineering, Ningbo University of Technology, Ningbo 315211, China; 2 Engineering Research Center of Industrial Construction in Civil Engineering of Zhejiang, Ningbo University of Technology, Ningbo 315211, China

**Keywords:** damage identification, static test, best achievable displacement, successive elimination, beam structure

## Abstract

To ensure the safe use of structures, it is essential to develop efficient damage identification techniques. In this paper, a brand-new approach to identifying structural deterioration based on static displacement is proposed. First, the relationship between the displacement variation and the damaged element is derived from the static response equations before and after damage. Subsequently, the optimal achievable displacement variation is defined to determine the damage location in the structure. A progressive elimination strategy is suggested to identify the real damaged parts and weed out the pseudo-damaged elements by measuring the distance between the measured and the best possible displacement variation. After determining the damage location, the corresponding damage extent can be calculated by a system of linear equations. The proposed approach has been tested on a beam structure and truss structure using simulated and experimental data. Compared with the existing static sensitivity method, the suggested method does not result in misjudgment and has higher identification accuracy. It has been demonstrated that the suggested approach is effective at locating and assessing the extent of structural damage.

## 1. Introduction

The identification of bridge structure damage has attracted extensive attention recently [1,2,3]. According to the type of data used, damage identification methods can be divided into dynamic methods and static methods. The fundamental concept behind these techniques is to determine the location and extent of structural damage by determining the damage characteristics from changes in static or dynamic factors. These damage detection methods can be subdivided into the static displacement-based method, the vibration frequency-based method, the vibration mode-based method, the dynamic flexibility-based method, the best achievable theory-based method, etc. 

For the static displacement-based method, Lee and Eun [4] proposed an analytical method for damage detection by using displacement curvature and deflection data expanded from the measured data. Jin et al. [5] proposed a static sensitivity method for damage detection by measuring the static displacement of the structure. By solving the static damage identification equation, the damage parameters of each element can be obtained and used to determine the location of damage in the structure. Song et al. [6] used the static strain data to obtain the corresponding static displacement for detecting structural damages. Ma et al. [7] employed wavelet analysis to diagnose structural defects using the static displacements of the structures. The fault position can be determined from the wavelet maxima lines and the fault severity can be assessed by the wavelet coefficients along the corresponding maxima lines. Wang et al. [8] proposed a technique for hanger fault diagnosis using static deflection change and the cable force of an arch bridge hanger. Numerical and experimental results indicated that their approach can successfully assess the location and reliability of faults in arch bridges.

For the vibration frequency-based method, Wang et al. [9] presented a damage detection method based on electromechanical admittances of multiple PZT patches and the cross-correlation coefficient. The damage sites and intensities of the plain concrete beam may be identified by monitoring the electromechanical admittance signals of each PZT over a range of frequencies. Dahak et al. [10] proposed a frequency contour method to detect the damage location and depth in a beam. The contour line was plotted using only the value of the changes in the measured natural frequencies and the vectors of the curvature mode shapes of the intact structure. Zhong et al. [11] developed a new approach based on auxiliary mass spatial probing using the spectral center correction method for the damage detection of beam-like structures. They discovered that fracture information for beam-like structural damage detection may be obtained from the derivatives of the natural frequency curve. Gillich et al. [12] found that relative frequency shifts are associated with structural defects and can be used to locate the cracks in a structure. Mousavi et al. [13] discovered that the instantaneous frequency of the first intrinsic mode function is related to the fault location in the structure. Mostafa et al. [14] employed the shape of the instantaneous frequency as a fault indicator to exclude the influence of the vehicle dynamics. They found that a high-resolution instantaneous frequency derived from the dynamic response could accurately identify structural defects.

For the vibration mode-based approach, Unger et al. [15] detected structural damage by minimizing the differences between the experimental mode shape and the corresponding analytical mode shape. A prestressed concrete beam experiment revealed that modal curvatures are very sensitive to local changes in bending stiffness close to the sensor position, but are insensitive to local changes farther from the measurement point. Tran-Ngoc et al. [16] proposed a damage detection method according to a combination of the artificial neural network (ANN) and cuckoo search (CS) algorithm. They discovered that, for localizing and quantifying structural damage, ANN-CS (ANN-CS) is more accurate than ANN and takes less time to compute. Altunışık et al. [17] carried out a detailed investigation on the modal parameter identification and vibration-based damage detection of a multiple-crack cantilever beam with a hollow circular cross-section. The disparities between the analytical data and the measured data for damage detection were reduced using the modal sensitivity approach based on Bayesian parameter estimation. To solve the problem of incomplete measurement, Yang and Peng developed the model condensation method [18] and proposed the modal condensation sensitivity [19] for the damage identification of beam structures. Pooya and Massumi [20] used the difference between mode shape curvature and the mode shape curvature estimation of damaged structures as an indicator to identify the damage location. 

For the dynamic flexibility-based method, Ahmadi-Nedushan and Fathnejat [21] presented a two-stage fault diagnosis approach based on the dynamic modal flexibility and the improved teaching–learning-based optimization technique. To find the defect location in the structure, He et al. [22] employed the deflection computed by the modal flexibility matrix. Using modal flexibility, Bernagozzi et al. [23] developed the data-driven criteria for structure-type classification to obtain the best approach for identifying structural defects. Liu et al. [24] developed a generalized flexibility matrix algorithm to address the issue of defect identification using incomplete mode shape data. Peng and Yang [25] carried out sensor placement and defect detection in steel truss bridges based on changes in generalized modal flexibility. 

As the best achievable theory-based method, Lim and Thomas [26] suggested the most viable eigenvector technique to ascertain the position and severity of damage in spatial truss structures. Despite the measurement inaccuracies unavoidably making the damaged location more challenging, their system works effectively. Zhao [27] and Ricci [28] further verified the best achievable eigenvector method with a shallow-arch structure and a 10-bay truss laboratory structure, respectively. Based on Bayesian inference, Prajapat and Ray-Chaudhuri [29] improved the detection accuracy of the best achievable eigenvector method under the interference of data noise. According to an experimental study involving a laboratory scale shear building and different stiffness modification scenarios, their algorithm is efficient enough to localize the stories with stiffness modifications.

In this work, a novel damage diagnosis approach is proposed using the best achievable displacement variation. The improvements to the suggested technique concentrate on the following two features when compared to the best feasible eigenvector methods currently used [26,27,28,29]. The first innovation is that the proposed method is based on static test displacement, while the existing methods are based on dynamic test modal data. Instead of performing dynamic tests, static tests have the advantage of being more accurate and having a simpler measuring approach. Moreover, the static test is more commonly used in the field of bridge engineering. The second innovation is that a successive elimination approach is proposed to determine the true damaged elements and exclude the pseudo-damaged elements more reliably. The suggested technique, which employs this successive elimination procedure, exceeds the current static sensitivity method in terms of accuracy and dependability. The proposed method is demonstrated by a numerical example and two experimental examples. The results of the three examples revealed that the proposed method can be successfully used for structural damage assessments. The basic framework of this work is as follows: Section 2 describes the theoretical basis, main formulas, and implementation steps of the best achievable displacement method for damage identification. In Section 3 and Section 4, the results of numerical verification and experimental verification are presented, respectively. Several conclusions are drawn in Section 5.

## 2. Theoretical Development

For a structure before and after damage, the static response equations can be expressed as:

(1) $$S_{u}d_{u}=f$$

(2) $$S_{d}d_{d}=f$$

(3) $$S_{d}=S_{u}-\Delta S$$

(4) $$d_{d}=d_{u}+\Delta d$$

where $S_{u}$ and $S_{d}$ are the stiffness matrices of the undamaged and damaged structure, $d_{u}$ and $d_{d}$ are the corresponding displacement vectors under the static load vector f, and ΔS and Δd are the changes in the stiffness matrix and the displacement owing to damage, respectively. Substituting Equations (3) and (4) into (1) gives

(5) $$S_{u}d_{u}+S_{u}\Delta d-\Delta Sd_{u}-\Delta S\Delta d=f$$

Substituting Equation (1) into (5), while excluding the high-order component, gives

(6) $$S_{u}\Delta d=\Delta Sd_{u}$$

Substituting Equation (1) into (6) gives

(7) $$S_{u}\Delta d=\Delta SS_{u}^{-1}f$$

Equation (7) can be rewritten as

(8) $$\Delta d=S_{u}^{-1}\Delta SS_{u}^{-1}f$$

In Equation (8), the stiffness change ΔS can be obtained by multiplying the sum of the elemental stiffness matrix by the damage coefficient as

(9) $$\Delta S=\sum_{i=1}^{N}\varepsilon_{i}S_{i},\;(0\leq \varepsilon_{i}\leq 1)$$

where $S_{i}$ is the i-th elementary stiffness matrix, $\varepsilon_{i}$ is the i-th elementary damage coefficient, N is the total number of elements. $\varepsilon_{i}=0$ denotes that the i-th element is undamaged. $\varepsilon_{i}$ equals 1, and less than 1 denotes that the damage to the i-th element is either complete or partial. Substituting Equation (9) into (8) yields

(10) $$\Delta d=\sum_{i=1}^{N}\varepsilon_{i}\xi_{i}$$

(11) $$\xi_{i}=S_{u}^{-1}S_{i}S_{u}^{-1}f$$

As mentioned before, $\varepsilon_{i}=0$ is valid for most undamaged elements. For convenience, the subsequent derivation process is carried out using three damaged elements as examples (other damage cases are also applicable). Without loss of generality, it is assumed that the x-th, y-th, and z-th elements are the potentially damaged elements. Thus, Equation (10) can be simplified by keeping only the damaged elements as

(12) $$\Delta d=\varepsilon_{x}\xi_{x}+\varepsilon_{y}\xi_{y}+\varepsilon_{z}\xi_{z}$$

(13) $$\xi_{x}=S_{u}^{-1}S_{x}S_{u}^{-1}f$$

(14) $$\xi_{y}=S_{u}^{-1}S_{y}S_{u}^{-1}f$$

(15) $$\xi_{z}=S_{u}^{-1}S_{z}S_{u}^{-1}f$$

The link between the displacement variation and the damaged elements is established by Equation (12). The physical meaning of Equation (12) is crucial and useful for damage localization. It is observed that Equation (12) is valid merely when Δd is a linear combination of the vectors $\xi_{x}$, $\xi_{y}$, and $\xi_{z}$ associated with the damaged elements. Equation (12) can be rewritten as

(16) $$\Delta d=\Omega_{x,y,z}\eta_{x,y,z}$$

(17) $$\Omega_{x,y,z}=[\xi_{x},\xi_{y},\xi_{z}]$$

(18) $$\eta_{x,y,z}={\left(\varepsilon_{x},\varepsilon_{y},\varepsilon_{z}\right)}^{T}$$

Based on Equation (16), the best feasible displacement variation can be defined to determine whether or not Δd is the linear combination of the column vectors of $\Omega_{x,y,z}$ as

(19) $$\zeta_{x,y,z}=\Omega_{x,y,z}\cdot \Omega_{x,y,z}^{+}\Delta d$$

where $\zeta_{x,y,z}$ is the best achievable displacement variation corresponding to the x-th, y-th, and z-th elements, and the Moore–Penrose inverse is denoted by the symbol “+”. If $\zeta_{x,y,z}$ is basically equal to Δd, it can be concluded that Δd is the linear combination of the column vectors of $\Omega_{x,y,z}$, and vice versa. In the meantime, the difference between Δd and $\zeta_{x,y,z}$ can be calculated by

(20) $$\delta_{x,y,z}={\left\|\Delta d-\zeta_{x,y,z}\right\|}_{2}$$

In which ${\left\|\cdot \right\|}_{2}$ denotes the 2-norm. For other element combinations, the corresponding δ can also be calculated by the above process. The minimum value of all δ will correspond to the damaged element combination. This means that the damage locations can be identified through searching the minimum value in all δ. For convenience, the relative distance $\delta_{x,y,z}^{\prime }$ can be defined for damage localization as

(21) $$\delta_{x,y,z}^{{}^{\prime }}=\frac{\delta_{x,y,z}}{\max(\delta )}$$

where max(δ) denotes the maximum value in all δ. If $\delta_{x,y,z}^{\prime }$ is equal to zero or extremely near to zero, it can be concluded that the elements related to $\delta_{x,y,z}^{\prime }$ are the damaged elements. Notice that the procedure described above may be applied to exclude those pseudo-damaged components. For instance, the best achievable displacement variation and the distance for the combination of the x-th and y-th elements can be calculated by

(22) $$\zeta_{x,y}=\Omega_{x,y}\cdot \Omega_{x,y}^{+}\Delta d$$

(23) $$\delta_{x,y}={\left\|\Delta d-\zeta_{x,y}\right\|}_{2}$$

If $\delta_{x,y}$ and $\delta_{x,y,z}$ are extremely close, it can be inferred that the z-th element is a pseudo-damaged element. In the end, the damage coefficients (i.e., damage extents) $\varepsilon_{x}$, $\varepsilon_{y}$ and $\varepsilon_{z}$ can be calculated easily from the linear Equation (16) as

(24) $$\eta_{x,y,z}=\Omega_{x,y,z}^{+}\Delta d$$

Note that a linear approximation that ignores the higher-order term is used in the derivation from Equation (5) to (6). Therefore, to obtain a more accurate damage coefficient, the acceleration formula derived from flexibility disassembly perturbation [30,31] should be employed as

(25) $$\varepsilon_{i}^{\prime }=\frac{\varepsilon_{i}}{1+\varepsilon_{i}}$$

To further assess the final damaged pieces, the calculated values of the damage coefficients can corroborate the findings of the damage localization based on the aforementioned best achievable hypothesis.

As a summary, a step-by-step description of the whole approach is illustrated as follows:

Step 1: Construct the finite element model of the structure to obtain the global stiffness matrix $S_{u}$ and the elementary stiffness matrices $S_{i}$ (i=1~N), and construct the finite element model of the structure. 

Step 2: Perform the static test on the structure before and after damage to obtain the displacement $d_{u}$, $d_{d}$ and the corresponding change Δd.

Step 3: For the combination of the possible damaged elements, use the best achievable theory described by Equations (16)–(23) to identify the genuine injured components and exclude the pseudo-damaged elements.

Step 4: Calculate the relevant damage coefficients (i.e., damage extents) for the detected damaged elements using Equations (24) and (25).

## 3. Verification by the Numerical Example

**Figure 1:**
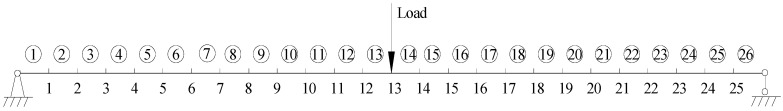
A beam structure.

The proposed damage identification method is illustrated numerically using the beam structure in Figure 1. To build the finite element model, the beam is evenly split into 26 segments using plane beam elements. Note that the finite element model is obtained by the MATLAB 2016b software, and the type of discretization element used is the Bernoulli–Euler plane beam element. The length of each segment is 0.1m. The physical and geometric parameters of this beam are as follows: Young’s modulus—30GPa; density—2500 kg/m^3^; cross-sectional area—0.21 m^2^; moment of inertia—8.575 × 10^−3^ m^4^. The decrease in the elastic modulus before and after damage simulates structural deterioration. It is assumed that the static vertical load of 4000 kN is applied at node 13, as shown in Figure 1. The analytical displacement data computed by the finite element models before and after damage are assumed to be the measured displacement data of the static test. The measurement error in practice is simulated by adding uniformly distributed random numbers to the analytical displacement data. 

**Figure 2:**
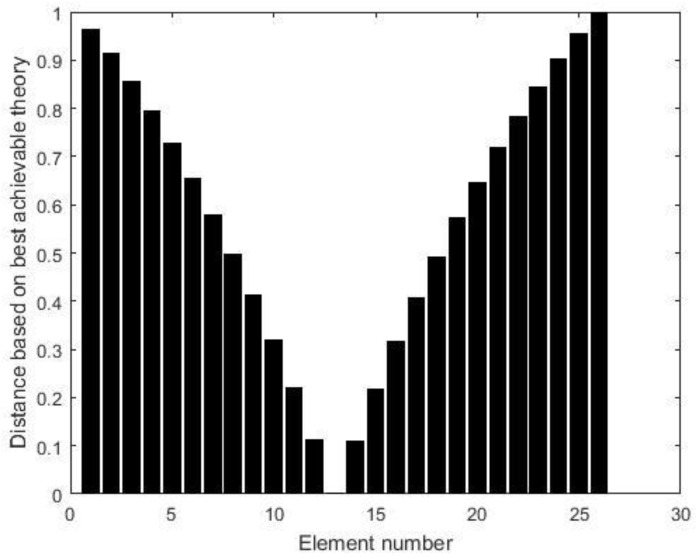
The distance $\delta_{i}^{\prime }$ (i=1~26) for each element when element 13 is damaged (no noise).

**Figure 3:**
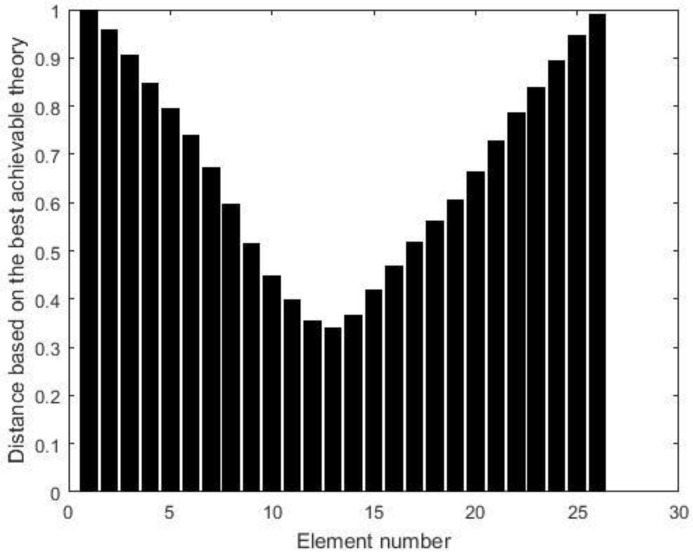
The distance $\delta_{i}^{\prime }$ (i=1~26) for each component when element 13 is damaged (3% noise).

**Figure 4:**
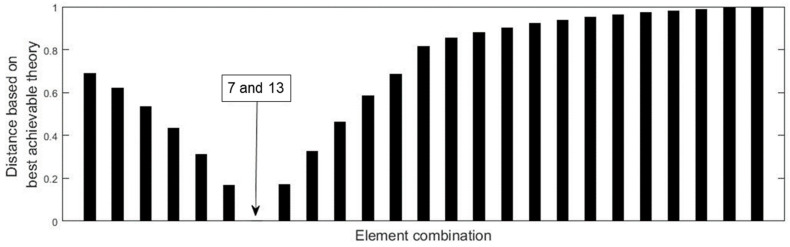
Distances for the element combinations when elements 7 and 13 are damaged (no noise).

**Figure 5:**
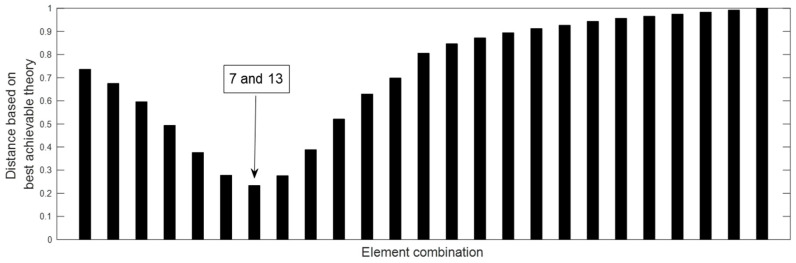
Distances for the combinations of components when elements 7 and 13 are damaged (3% noise).

Two damage scenarios are then simulated to verify the proposed method. The first damage scenario estimates a 20% reduction in the elastic modulus of element 13. Using the displacement data without noise, Figure 2 presents all relative distances $\delta_{i}^{\prime }$ (i=1~26) for each element based on the above best achievable technique. According to Figure 2, it can be discovered that only $\delta_{13}^{\prime }=0$ and the others are greater than zero. As a result, element 13 is the only one that can be determined to be damaged, and its damage extent can be calculated as $\varepsilon_{13}=0.2$, which is exactly the value that was presumed. When 3% noise is added to the displacement data, Figure 3 presents all the relative distances $\delta_{i}^{\prime }$ (i=1~26) for each element based on the above best achievable technique. Figure 3 reveals that $\delta_{13}^{\prime }$ is the minimum value for all $\delta_{i}^{\prime }$. This means that element 13 can be recognized as the damaged element and its damage extent can be calculated as $\varepsilon_{13}=0.1943$, which is close to the assumed value 0.2. The second damage scenario implies that the elastic moduli of elements 7 and 13 are reduced by 15% and 20%, respectively. Figure 4 displays the relative distances for a selection of element combinations using noise-free displacement data. From Figure 4, it can be found that only the distance associated with the combination of components 7 and 13 is equal to zero. This indicates that elements 7 and 13 can be determined to be the damaged elements. Their damage extents can be calculated as $\varepsilon_{7}=0.15$ and $\varepsilon_{13}=0.2$, which are exactly the assumed values. Figure 5 illustrates the relative distances for several element combinations using displacement data with 3% noise and the above-mentioned best method. From Figure 5, it can be found that the combination of elements 7 and 13 corresponds to the smallest of all calculated distances. Thus, elements 7 and 13 can be determined to be the damaged elements, and the damage extents can be calculated as $\varepsilon_{7}=0.1573$ and $\varepsilon_{13}=0.2103$, which are close to the assumed values (0.15 and 0.2). The findings of this numerical example demonstrate that the suggested technique may successfully locate and assess damage in the beam structure.

## 4. Verification by the Experimental Example

### 4.1. A Channel Steel Beam

The proposed method is further validated using an experimental beam carried out by Le et al. [32]. This experimental beam is separated into five segments, and the deflection information under mid-span stress is gathered using six displacement meters. The total length of the channel steel beam is 2475 mm. The finite element model of this beam is also achieved using MATLAB software, and the Bernoulli–Euler plane beam element is the discretization element of choice. The damage is simulated by cutting a part of the steel beam, and the cutting position is 1175 mm away from the left support. The segment number corresponding to the damage location is three. The reference [32] describes the specifics of the static test procedure, as well as the obtained deflection data. 

**Figure 6:**
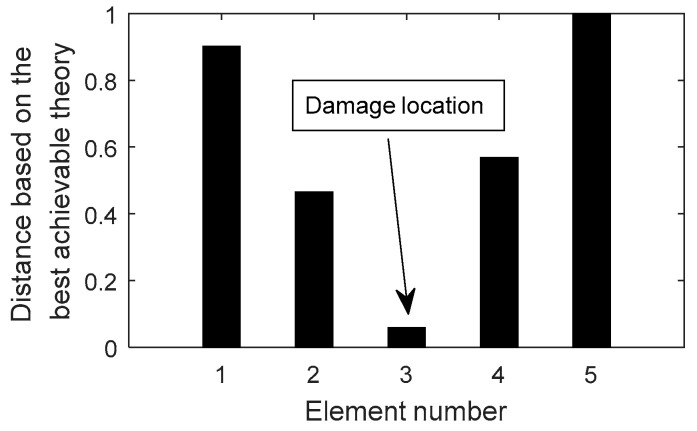
The distance $\delta_{i}^{\prime }$ (i=1~5) for the experimental beam when segment 3 is damaged.

Using the measured deflection data, Figure 6 presents all relative distances $\delta_{i}^{\prime }$ (i=1~5) for each segment based on the aforesaid best feasible method. According to Figure 6, $\delta_{3}^{\prime }$ is the minimum value for all $\delta_{i}^{\prime }$. It can be concluded that segment 3 can be effectively identified as the site of damage in the structure using the suggested method.

### 4.2. A Steel Truss Structure

**Figure 7:**
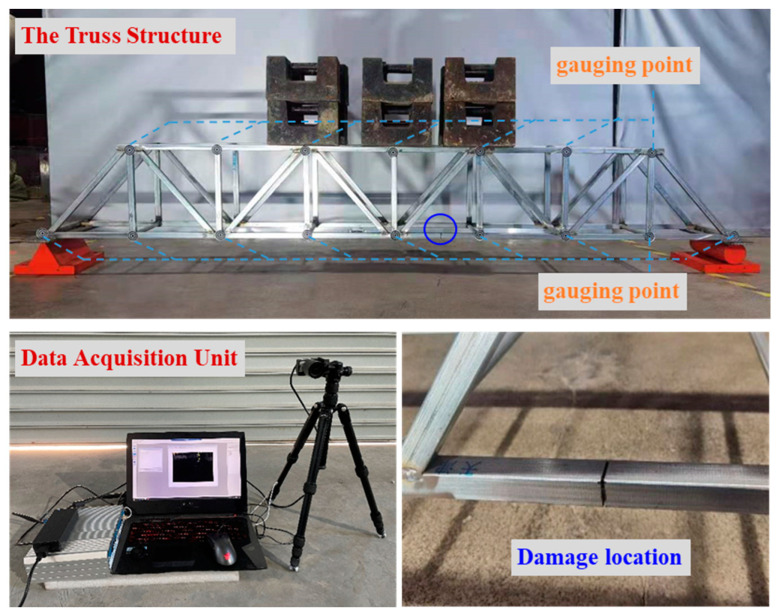
A steel truss structure.

**Figure 8:**
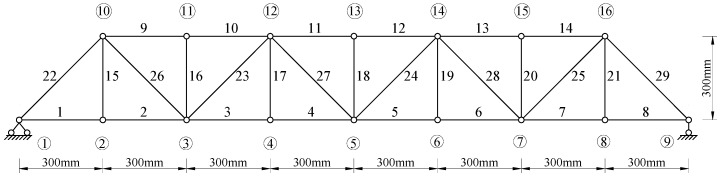
Finite element model of the steel truss structure.

A steel truss construction is constructed, as shown in Figure 7, to further validate the presented algorithm. The particular properties of this structure are listed in Table 1. Using the MATLAB software, Figure 8 presents the plane finite element model by considering the symmetry of the structure. The plane bar element is the kind of discretization element that is used. The structure is statically loaded by stacking several weights, each weighing 25 kg. Cutting 40% of the cross-section of bar element 5 causes structural damage, as illustrated in Figure 7. Each node in the truss construction has its static displacement data collected using a high-precision optical measuring device both before and after damage. 

**Figure 9:**
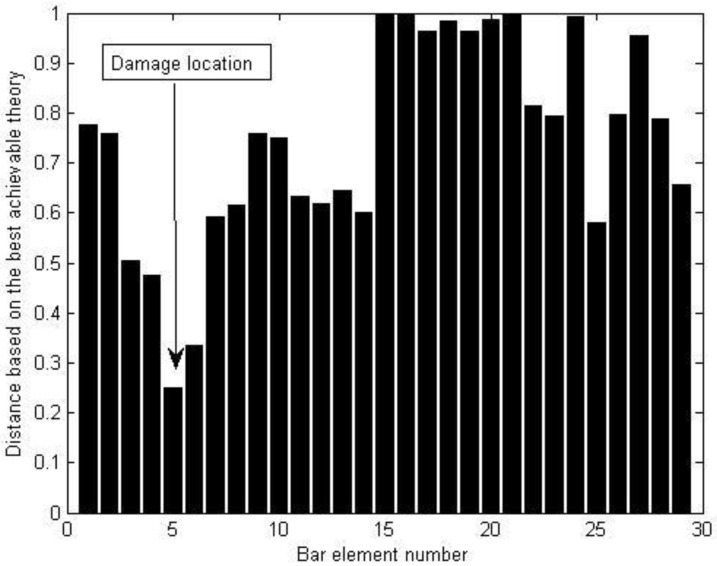
The distance $\delta_{i}^{\prime }$ (i=1~29) for the experimental truss structure when bar element 5 is damaged.

**Figure 10:**
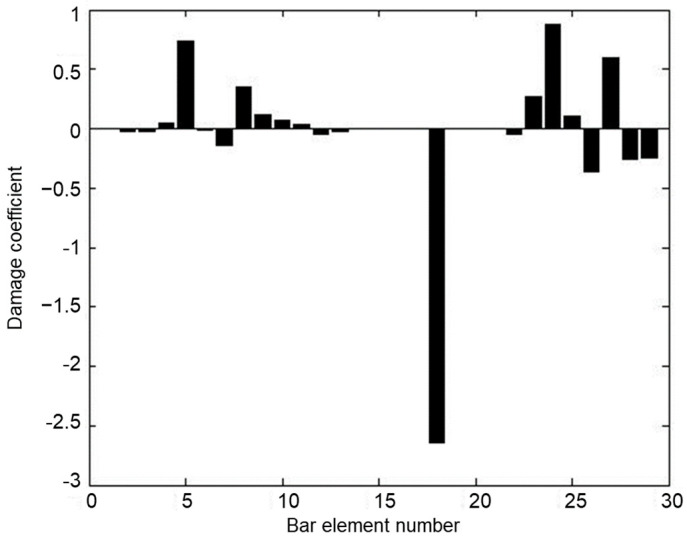
The damage coefficients obtained by the existing static sensitivity method.

Using the measured displacement data of the undamaged and damaged structure, Figure 9 provides all relative distances $\delta_{i}^{\prime }$ (i=1~29) for each bar element obtained by the proposed best achievable algorithm. From Figure 9, it can be noticed that $\delta_{5}^{\prime }$ is the minimum value in all $\delta_{i}^{\prime }$. It can be concluded that bar element 5 can be successfully detected as the damage location in the structure by the proposed method. Furthermore, the corresponding damage coefficient (i.e., damage extent) of bar element 5 can be calculated by Equations (24) and (25) as $\varepsilon_{5}^{\prime }$ = 0.3962, which is almost identical to the actual amount of 40%. To illustrate the advantage of this method, the calculation result of the existing static sensitivity method in reference [5] is also displayed in Figure 10 for comparison with the result of this method. From Figure 10, it can be observed that the existing static sensitivity method is unable to identify the unique damage location of element 5. The bar elements 8, 23, 24, and 27 are incorrectly identified as the damaged elements. In addition, the calculated damage coefficient of element 5 in Figure 10 is 0.7323, which deviates more from the true value compared to the calculated result of the proposed method. This demonstrates that the proposed method has more dependability and calculation accuracy.

## 5. Conclusions

In this paper, a novel method of structural damage identification based on the best achievable theory is proposed. According to the static response equations before and after damage, the displacement variation can be expressed as a linear combination of a series of vectors associated with the damaged elements. In this study, the distance between the measured and the best practicable displacement variation is calculated to determine the best achievable displacement variation for detecting the damage location using the best achievable theory. For the multi-damage case, a successive elimination approach is proposed to determine the true damaged elements and exclude the pseudo-damaged elements. When the best achievable theory is used to determine the damage location, the corresponding damage extents can be easily calculated by solving the linear equations. The suggested approach has been effectively validated on a beam structure and truss structure using simulated and experimental data. Analyzing the numerical and experimental results allows for the following conclusions: (1) Compared with the dynamic method, the proposed method only requires the measurement of the static displacement parameters of the structure under gravity loads. The measurement process is simpler and more accurate than the dynamic method. (2) The damaged element or its combination will correspond to the minimum value of the distances between the measured and the optimal achievable displacement variation. This principle can be successfully used to determine the location of damage in the structure. (3) The fault extent may be accurately calculated using the Moore–Penrose inverse and the acceleration formula once the fault location has been identified through the best feasible theory. (4) Compared with the existing static sensitivity method, the proposed method has higher reliability and calculation accuracy. The suggested approach may effectively reduce the potential for error in the current static sensitivity technique and can obtain damage coefficient values closer to the true values. It has been indicated that the proposed method may be an effective tool in structural damage detection in practice. Future research directions could focus on the following aspects. To further assess the reliability of this method, the first step is to apply it to real engineering issues. The second is to discuss the influence of the number and location of static displacement observation points on the identification accuracy of this method. Based on this, the economical layout of displacement observation points can be studied to facilitate the implementation of this method. The third is to apply this technique to several kinds of structures, such as composite structures, plate structures, and arch structures.

## Figures and Tables

**Table 1:** Geometric and material properties.

Parameter	Material	Modulus of Elasticity	Poisson’s Ratio	Mass Density	Section	Width	Depth	Thickness
Value	Steel	2.06 × 10^5^ MPa	0.3	7850 kg/m^3^	Square tube	20 mm	20 mm	1.2 mm

## Data Availability

Not applicable.
